# Supplementary material for: PREDICTD PaRallel Epigenomics Data Imputation with Cloud-based Tensor Decomposition
Source: Nat Commun. 2018 Apr 11;9:1402. doi: 10.1038/s41467-018-03635-9 (PMC5895786; doi:10.1038/s41467-018-03635-9)
Supplement: Supplementary file 1 — Supplementary Information(PDF 14720 kb) [file 41467_2018_3635_MOESM1_ESM.pdf]

Supplementary Information

Durham, et al. PREDICTD PaRallel Epigenomics Data Imputation with Cloud-based Tensor Decomposition

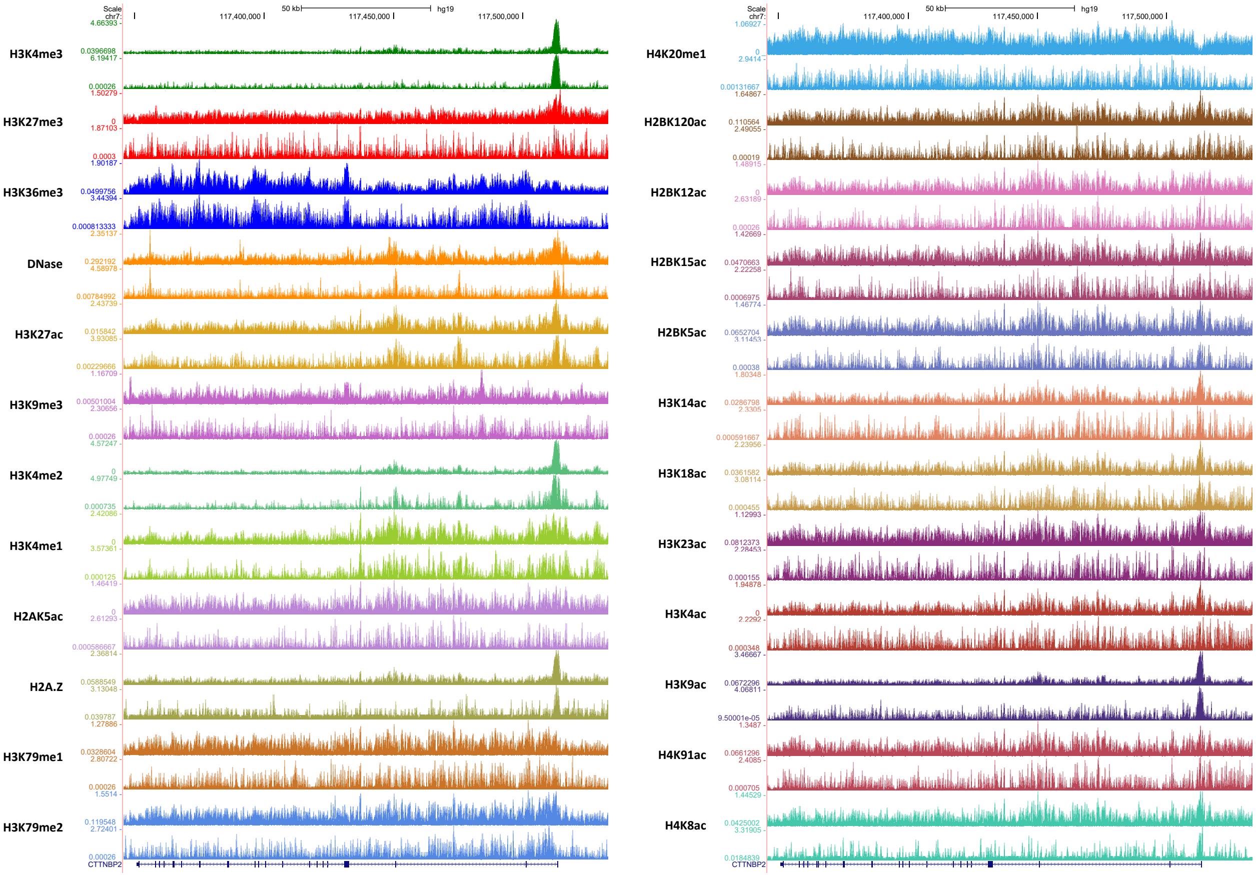

Supplementary Figure 1: **Example tracks show accuracy and diversity of imputed signals.** Tracks showing paired PREDICTD (top) and observed (bottom) data for the H1 Cell Line cell type, which is one of only three cell types for which observed data are available for all assays. The signal is variance stabilized with the inverse hyperbolic sine transform, and the tracks are auto-scaled by the genome browser to highlight the shape of the signal.

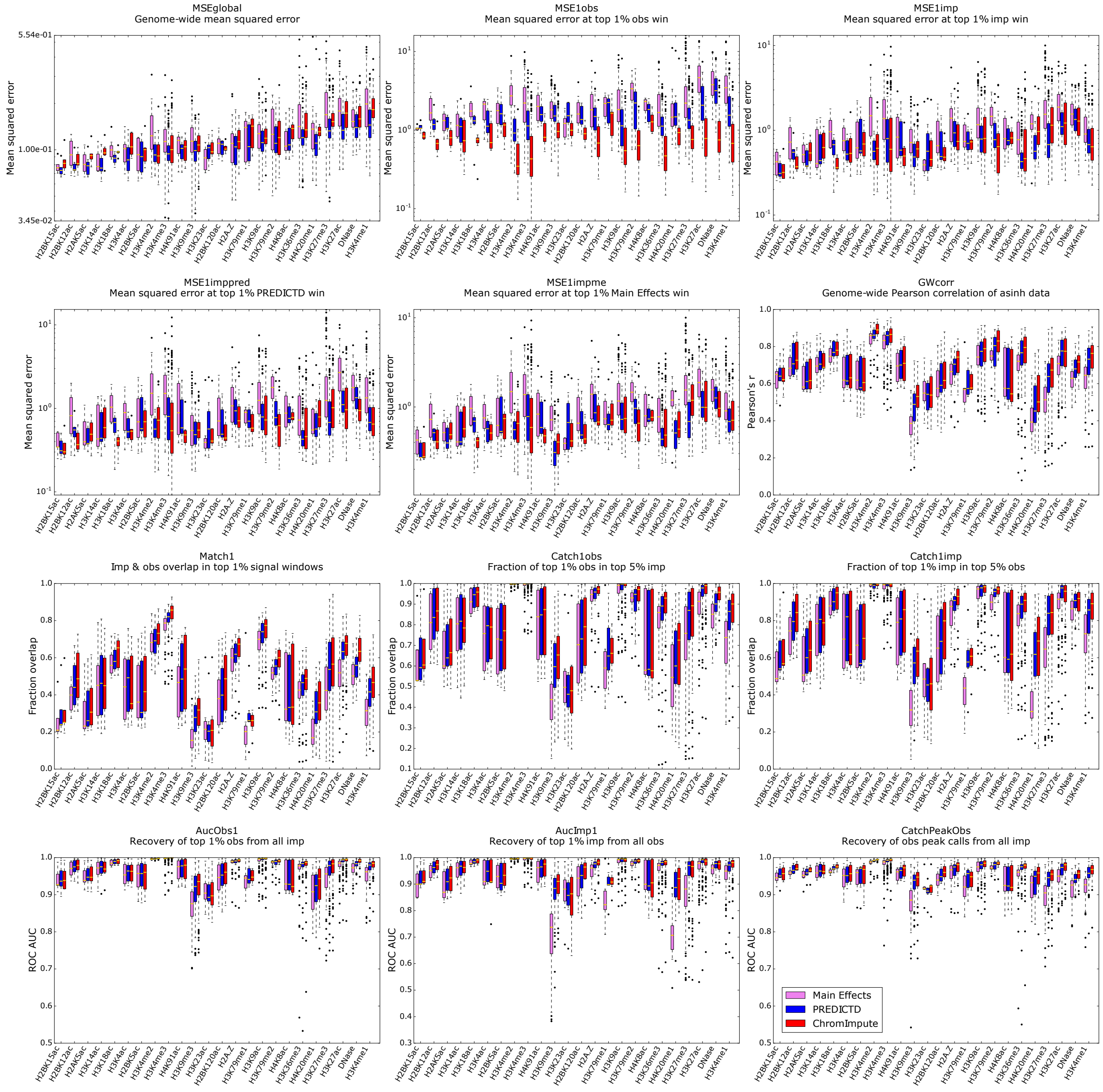

Supplementary Figure 2: **Plots for all quality measures evaluating the performance of three imputation methods.** Box plots describe the distribution of quality measure values for Main Effects (pink), PREDICTD (blue), and ChromImpute (red) for each assay. Each plot shows a different quality measure, and for each distribution of scores the box shows the inter-quartile range (IQR), whiskers show 1.5 times the IQR, and flier points show scores for individual experiments that are outliers. The median is indicated by a horizontal gold line on each box plot.

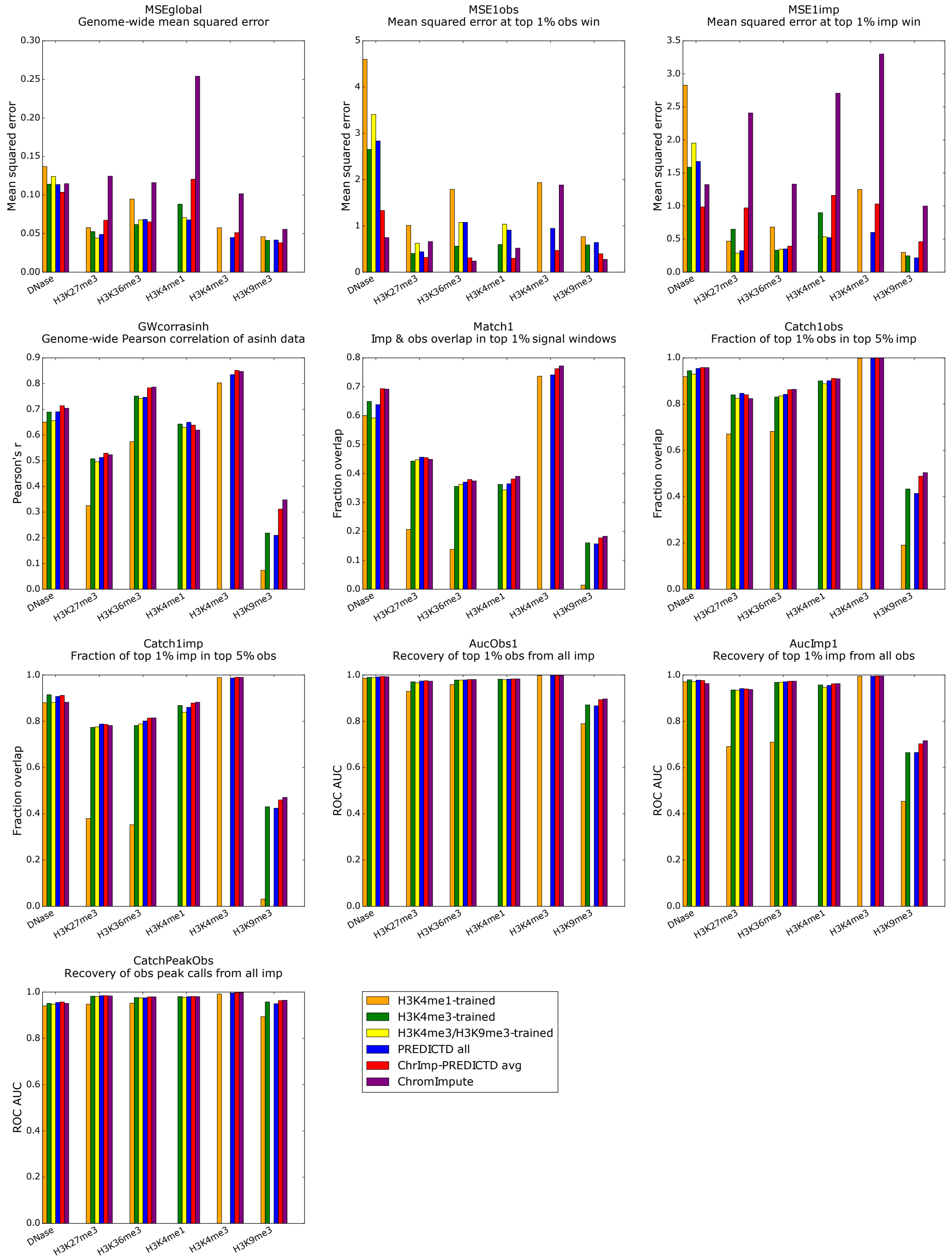

Supplementary Figure 3: **PREDICTD can still impute accurate data for "CD3 Primary Cells from Cord Blood" even with only one or two assays included in the training set.** Each plot shows the data for a different quality measure, and the bars compare the results of running PREDICTD with just H3K4me1 (orange), just H3K4me3 (green), or H3K4me3 and H3K9me3 (yellow) data included in the training set for "CD3 Primary Cells from Cord Blood". The quality of these imputation results are compared with the full PREDICTD imputation results (blue), the average of PREDICTD and ChromImpute (red), and ChromImpute alone (purple).

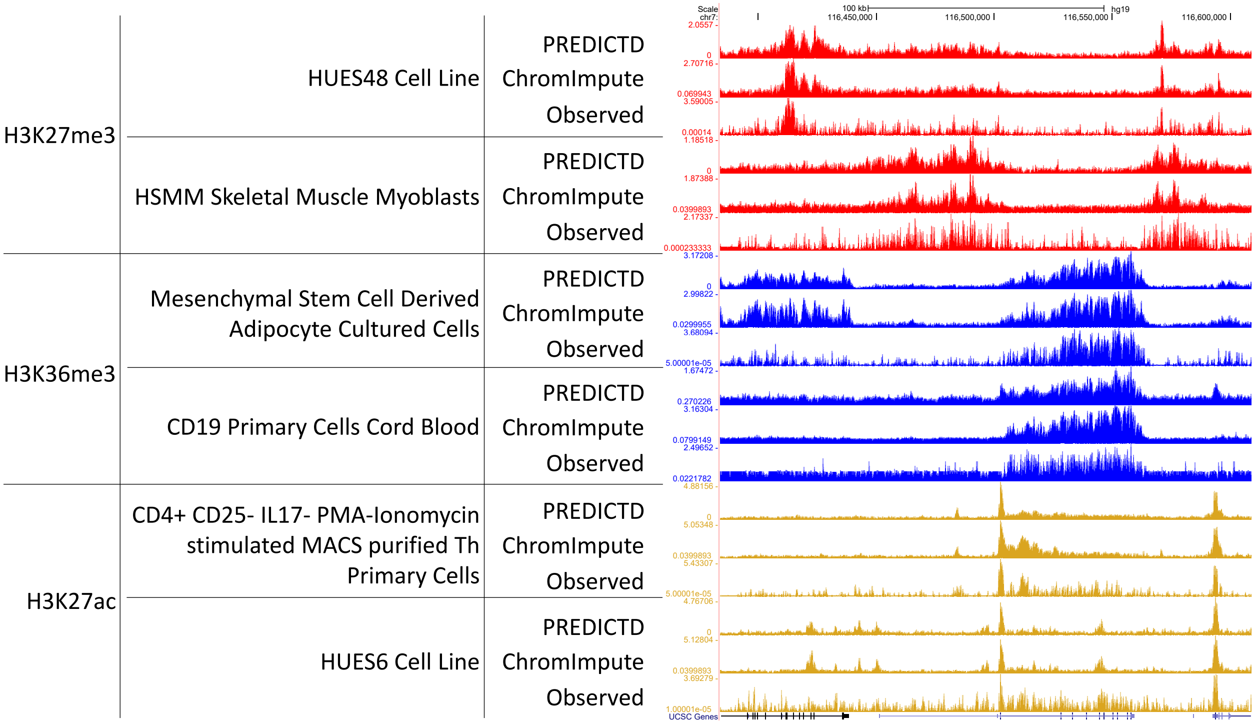

Supplementary Figure 4: **Selected tracks comparing PREDICTD imputed signal, ChromImpute imputed signal, and observed signal for three assays and six cell types.**

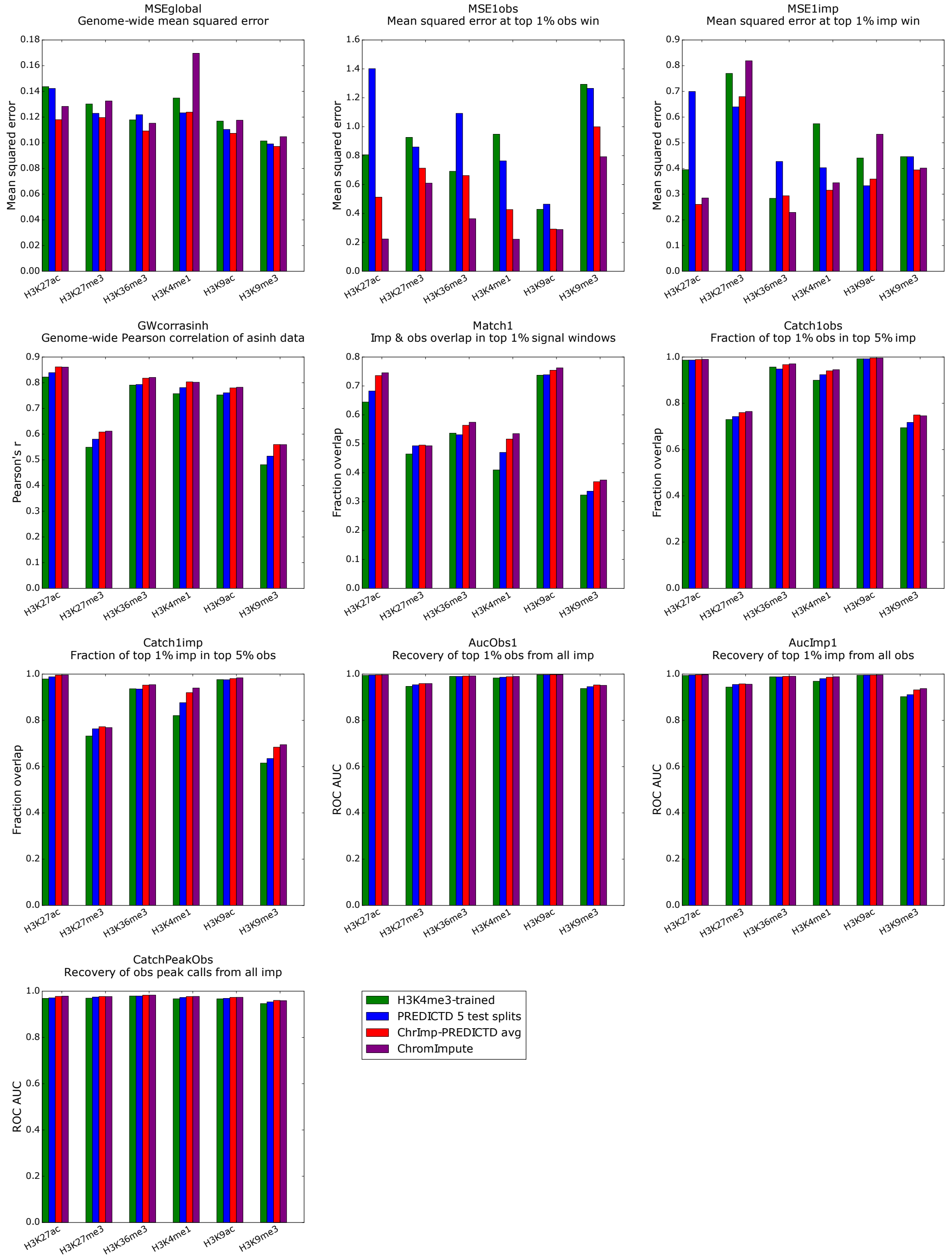

Supplementary Figure 5: **PREDICTD can still impute accurate data for "Brain Anterior Caudate" even with only the H3K4me3 assay included in the training set.** Each plot shows the data for a different quality measure, and the bars compare the results of running PREDICTD with just H3K4me3 (green) data included in the training set for "Brain Anterior Caudate". The quality of these imputation results are compared with the full PREDICTD imputation results (blue), the average of PREDICTD and ChromImpute (red), and ChromImpute alone (purple).

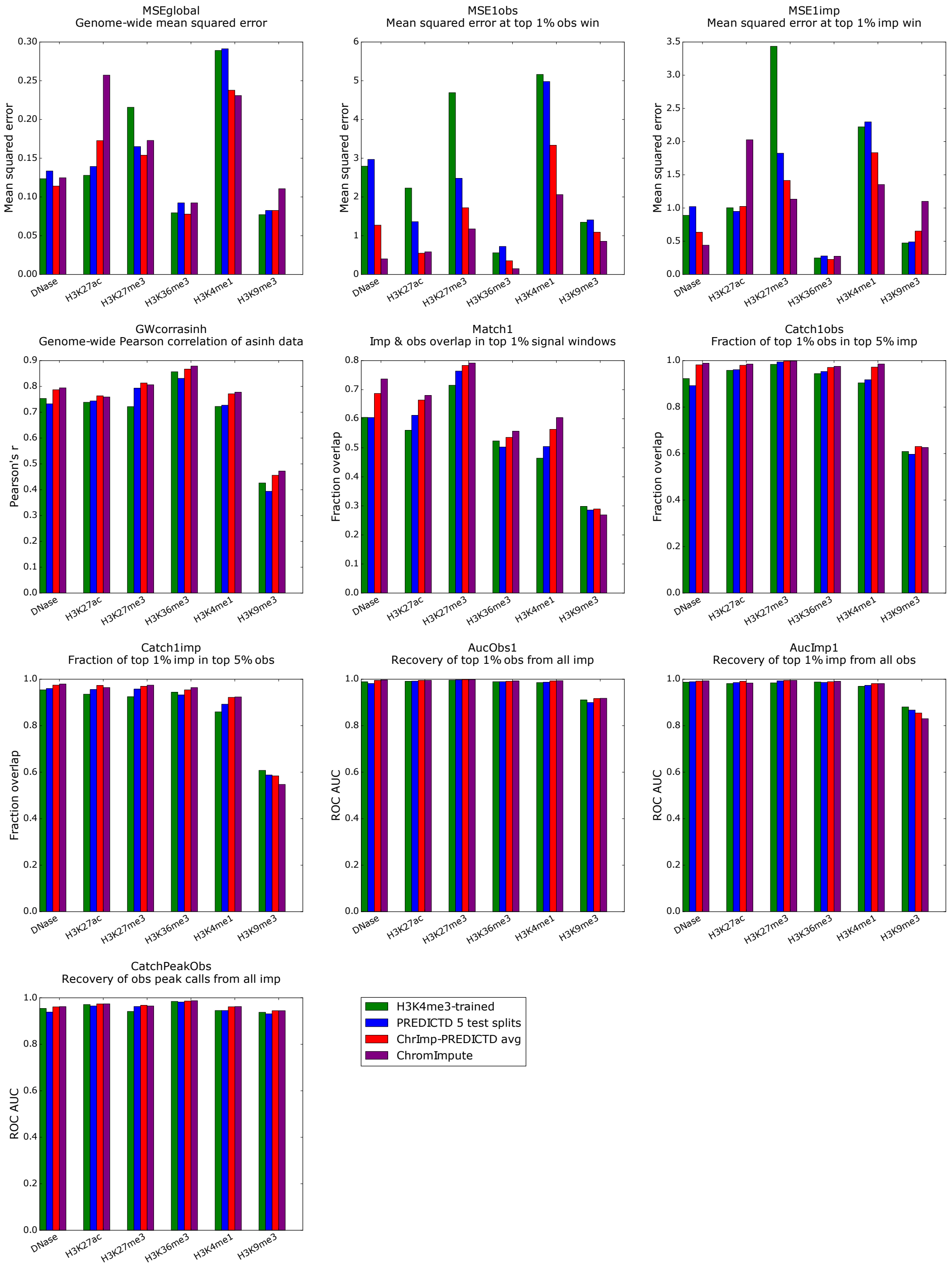

Supplementary Figure 6: **PREDICTD can still impute accurate data for "Fetal Muscle Trunk" even with only the H3K4me3 assay included in the training set.** Each plot shows the data for a different quality measure, and the bars compare the results of running PREDICTD with just H3K4me3 (green) data included in the training set for "Fetal Muscle Trunk". The quality of these imputation results are compared with the full PREDICTD imputation results (blue), the average of PREDICTD and ChromImpute (red), and ChromImpute alone (purple).

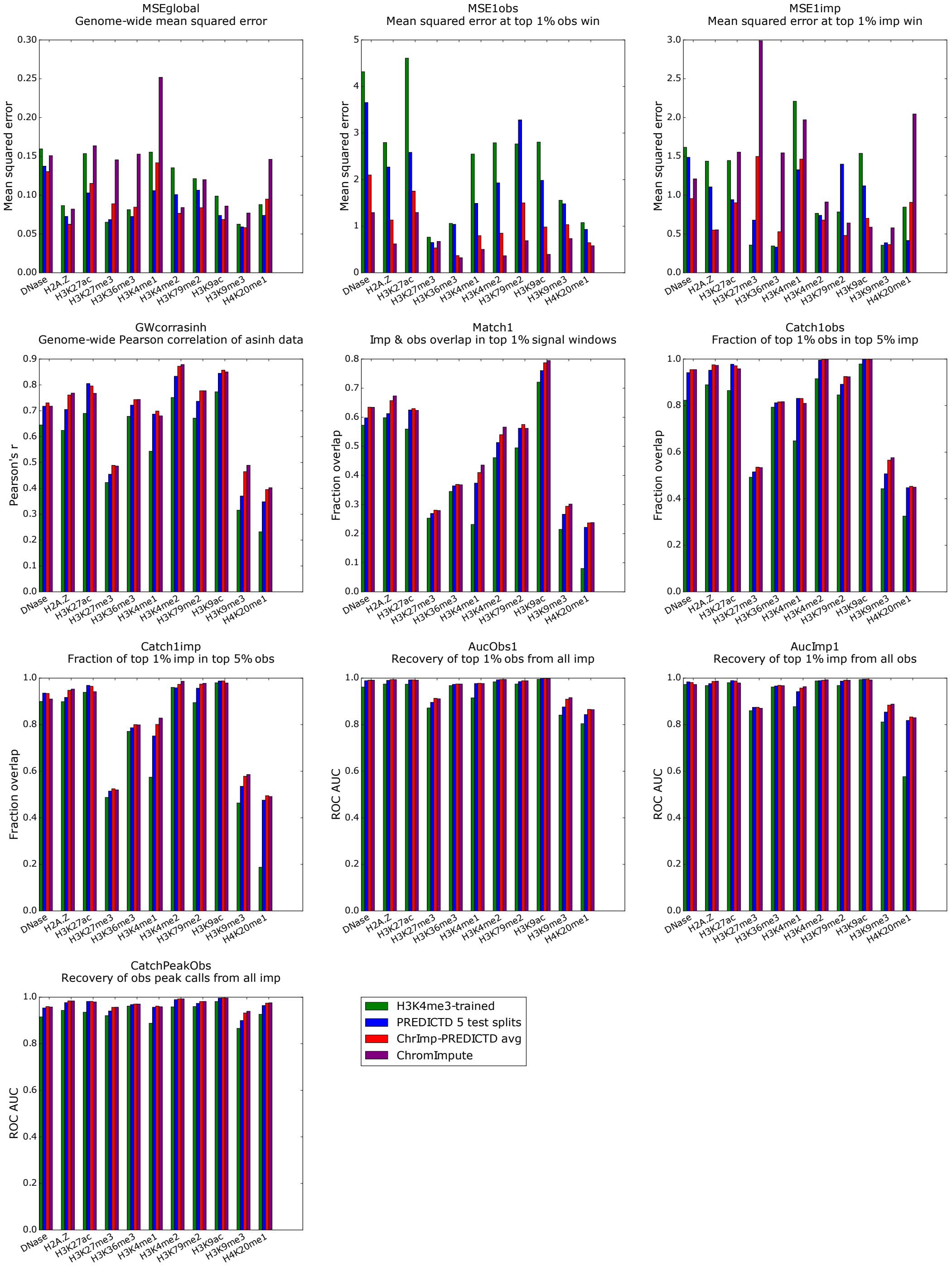

Supplementary Figure 7: **PREDICTD can still impute accurate data for "GM12878 Lymphoblastoid" even with only the H3K4me3 assay included in the training set.** Each plot shows the data for a different quality measure, and the bars compare the results of running PREDICTD with just H3K4me3 (green) data included in the training set for "GM12878 Lymphoblastoid". The quality of these imputation results are compared with the full PREDICTD imputation results (blue), the average of PREDICTD and ChromImpute (red), and ChromImpute alone (purple).

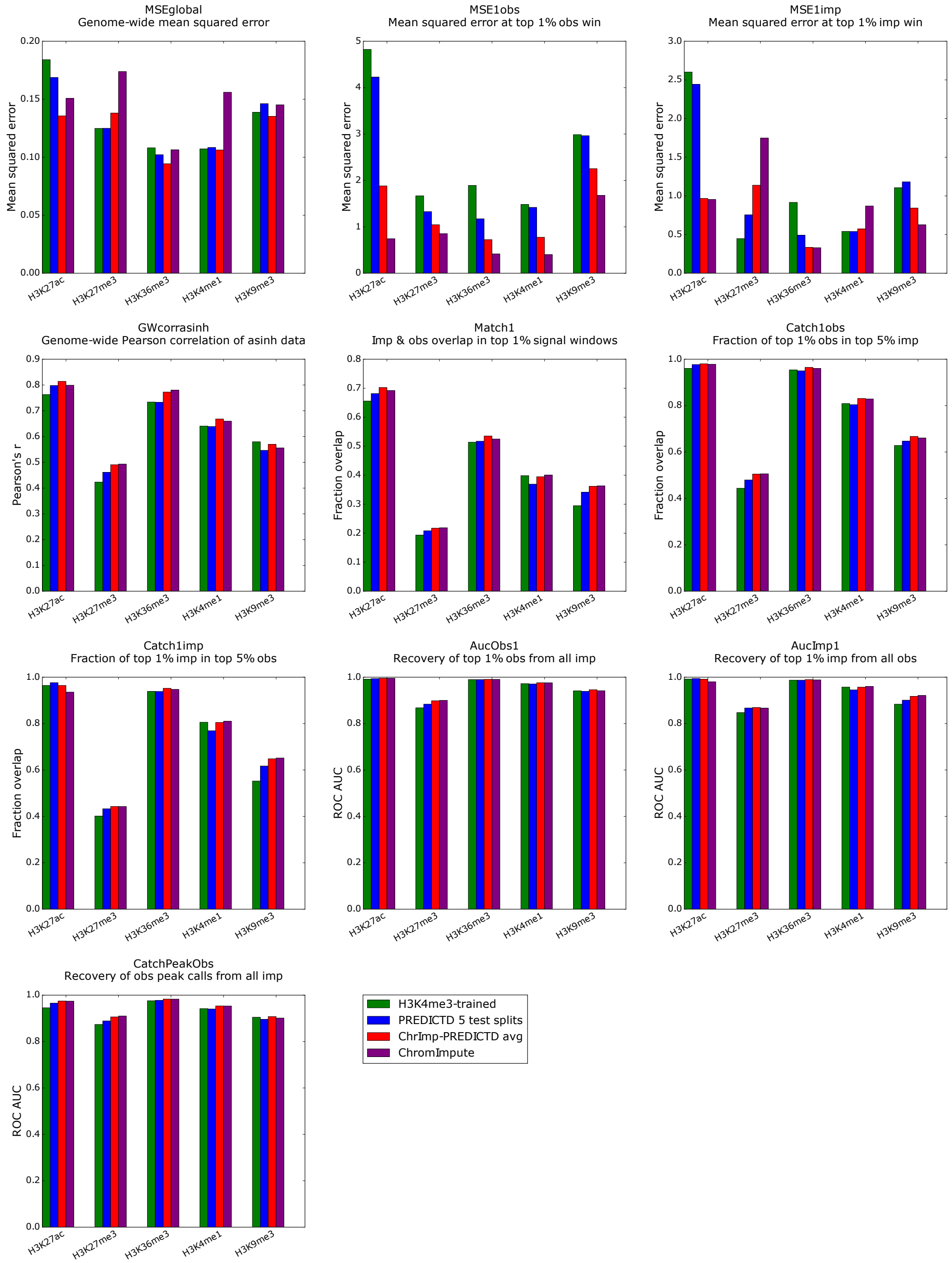

Supplementary Figure 8: **PREDICTD can still impute accurate data for "Lung" even with only the H3K4me3 assay included in the training set.** Each plot shows the data for a different quality measure, and the bars compare the results of running PREDICTD with just H3K4me3 (green) data included in the training set for "Lung". The quality of these imputation results are compared with the full PREDICTD imputation results (blue), the average of PREDICTD and ChromImpute (red), and ChromImpute alone (purple).

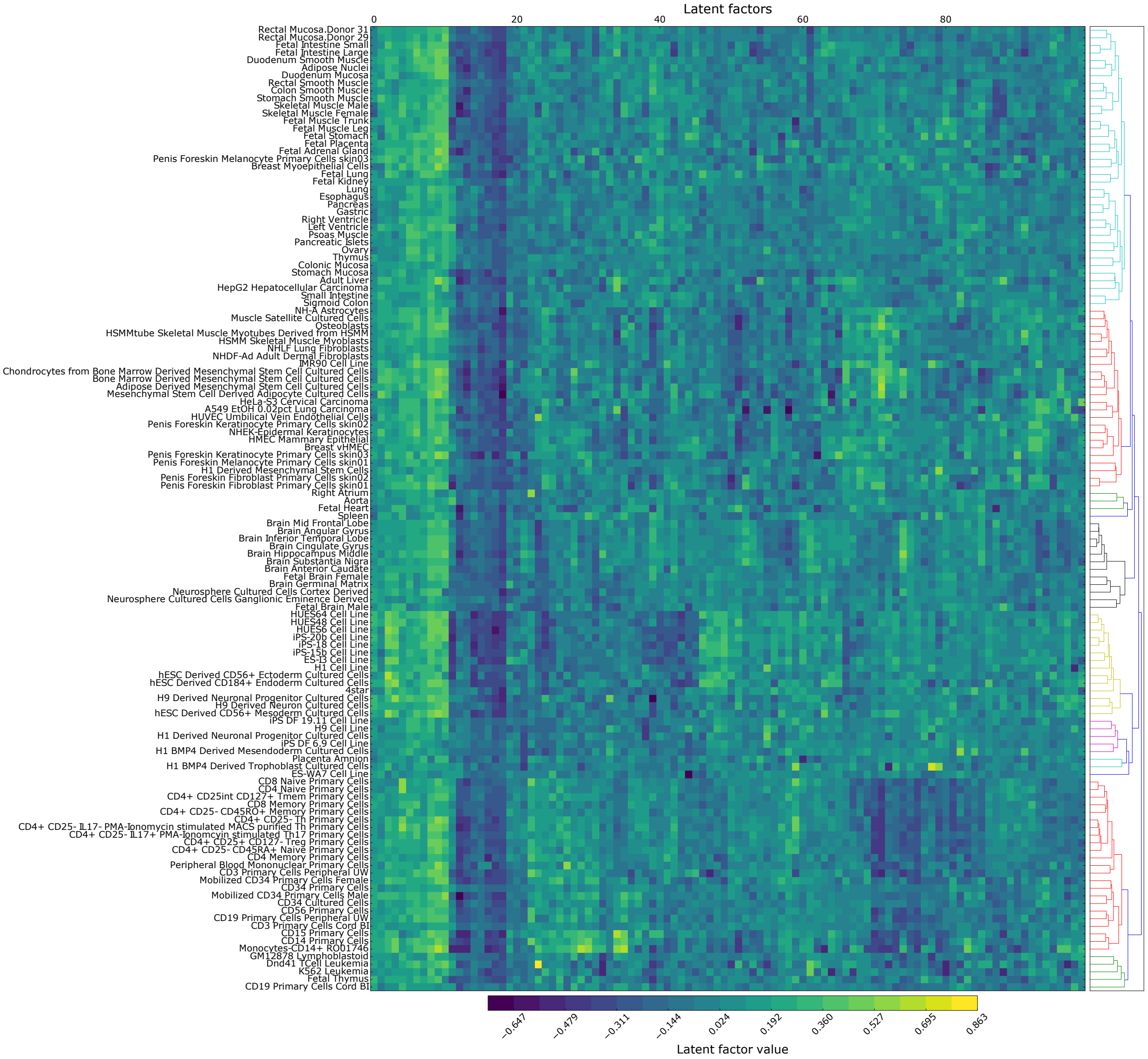

Supplementary Figure 9: **PREDICTD parameter values can distinguish among cell types.** Hierarchical clustering of all cell types based on parameter values from the cell type factor matrix. For legibility, Fig. 3a shows only the lower 62 rows of this figure.

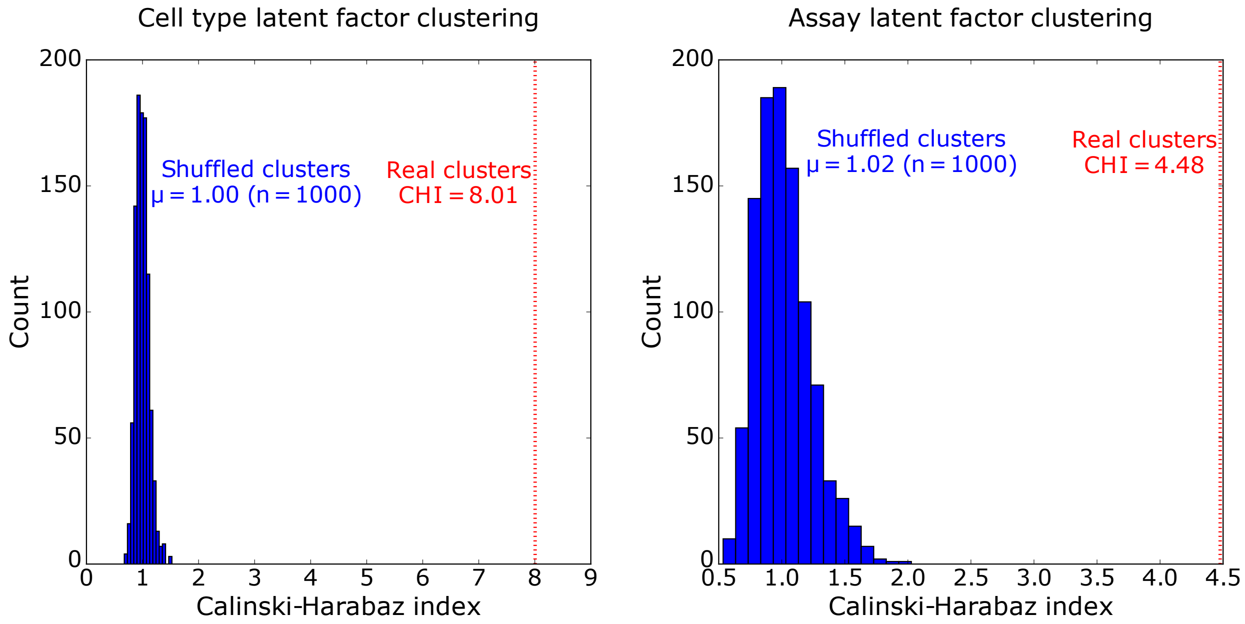

Supplementary Figure 10: **Hierarchical clustering of cell types and assays by latent factor parameter values is highly non-random.** Separation of clusters was tested using the Calinski-Harabaz Index after randomly assigning cluster identities to cell types and assays. This was repeated 1000 times and compared with the separation achieved by the true latent factor clustering. A higher value on the Calinski-Harabaz Index indicates that the clusters are denser and better-separated. Linkage trees were cut at eight clusters for cell types and four clusters for assays.

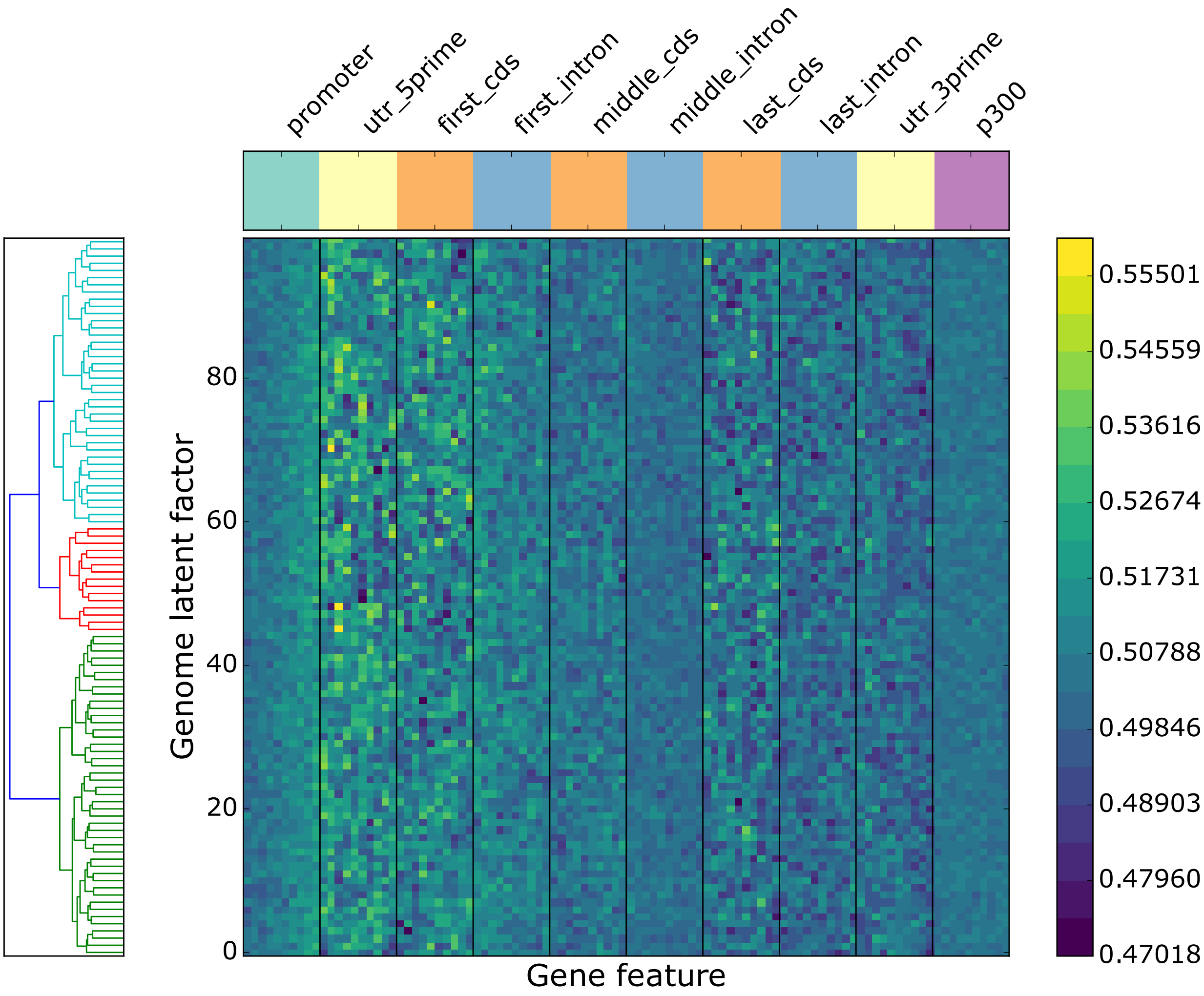

Supplementary Figure 11: **Patterns in the average latent factor values at different classes of genomic elements are non-random.** The same analysis was completed as in Fig. 3c, but after randomly permuting the latent factors at each genomic position. Randomly permuted latent factors do not show distinct patterns at different genomic elements.

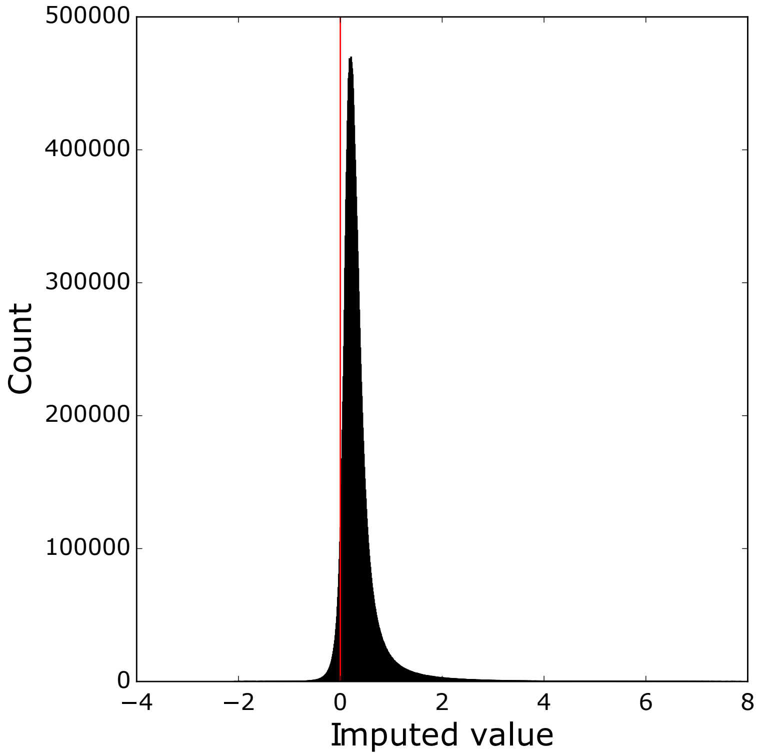

Supplementary Figure 12: **Most imputed values are positive despite allowing model parameters to have negative values.** The cell type, assay, and genome parameters from one of the 48 models trained on the ENCODE Pilot Regions plus non-coding human accelerated regions were used to impute values for 100000 randomly selected genomic positions, and these imputed values are plotted here as a histogram.

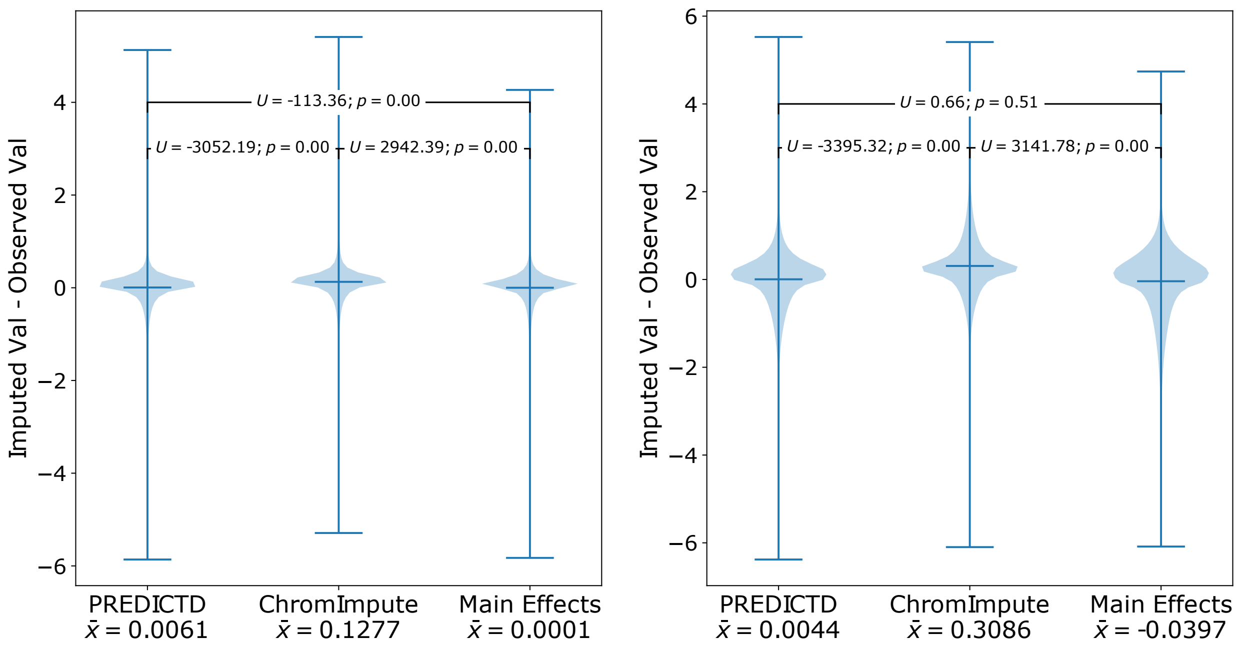

Supplementary Figure 13: **The error distribution of ChromImpute values is more positive than that of PREDICTD.** This means that on average ChromImpute tends to over-estimate signal amplitude compared to PREDICTD. Error distributions for each model were compared with the Mann-Whitney U test, and the sample mean is reported for each model on the x-axis. Violin plots show the distribution of error values, with a horizontal line at the mean and whiskers indicating the extrema. **a.** Random sample of 100,000 genomic positions. **b.** Top 100,000 genomic positions by summing all observed data values at each position.

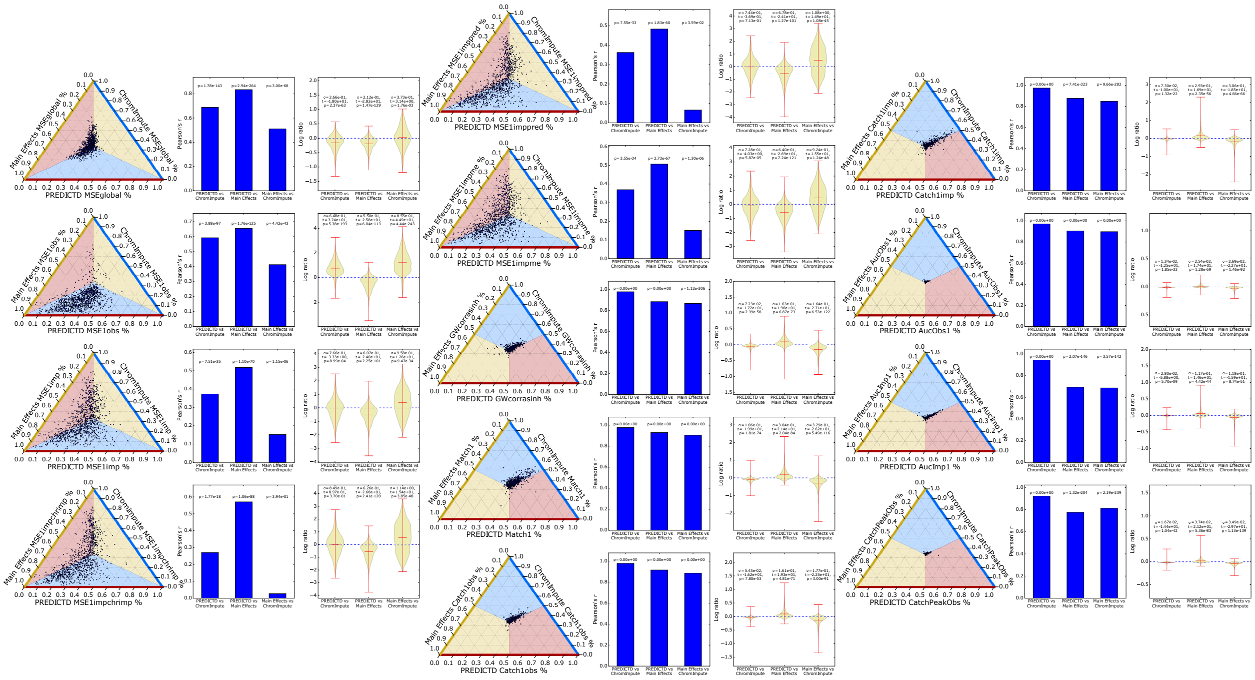

Supplementary Figure 14: **Comparison of all quality measures for PREDICTD, ChromImpute, and Main Effects models.** The first plot in each triplet is the ternary plot, as described in Fig. 4, the second is the Pearson correlation between the quality measure values for each pair of models, and the third is the distribution of the natural log fold-change in quality measure value between corresponding experiments in pairs of models. Each violin plot shows the distribution of the natural log fold-change values, with a horizontal line at the mean and whiskers indicating the extrema. Note that the correlation of PREDICTD with ChromImpute is always higher than the correlation between Main Effects and ChromImpute, indicating that PREDICTD tends to agree more with ChromImpute than Main Effects does. In addition, the mean log fold-change between PREDICTD and ChromImpute is always either closer to zero than the log fold-change between Main Effects and ChromImpute, indicating more comparable quality measure values between PREDICTD and ChromImpute, or the mean log fold-change indicates stronger performance by PREDICTD (MSEglobal, MSE1imp, and MSE1impme) than ChromImpute. In all, the quality measures show that PREDICTD performs very similarly to ChromImpute, and more so than Main Effects does.

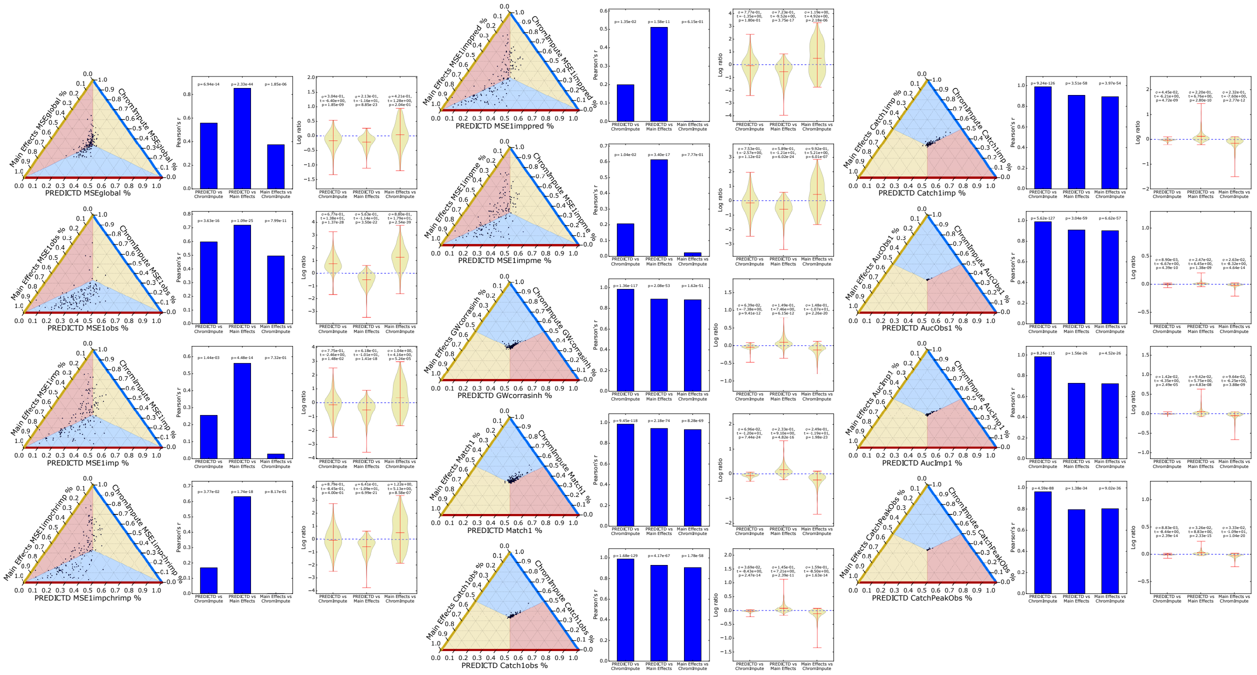

Supplementary Figure 15: **Comparison of all quality measures for PREDICTD, ChromImpute, and Main Effects models on just the 153 held out final test experiments that were not used in hyperparameter tuning.** The first plot in each triplet is the ternary plot, as described in Fig. 4, the second is the Pearson correlation between the quality measure values for each pair of models, and the third is the distribution of the natural log fold-change in quality measure value between corresponding experiments in pairs of models. Each violin plot shows the distribution of the natural log fold-change values, with a horizontal line at the mean and whiskers indicating the extrema. Note that the correlation of PREDICTD with ChromImpute is always higher than the correlation between Main Effects and ChromImpute , indicating that PREDICTD tends to agree more with ChromImpute than Main Effects does. In addition, the mean log fold-change between PREDICTD and ChromImpute is always either closer to zero than the log fold-change between Main Effects and ChromImpute, indicating more comparable quality measure values between PREDICTD and ChromImpute, or the mean log fold-change indicates stronger performance by PREDICTD (MSEglobal, MSE1imp, and MSE1impme) than ChromImpute. In all, the quality measures show that PREDICTD performs very similarly to ChromImpute, and more so than Main Effects does.

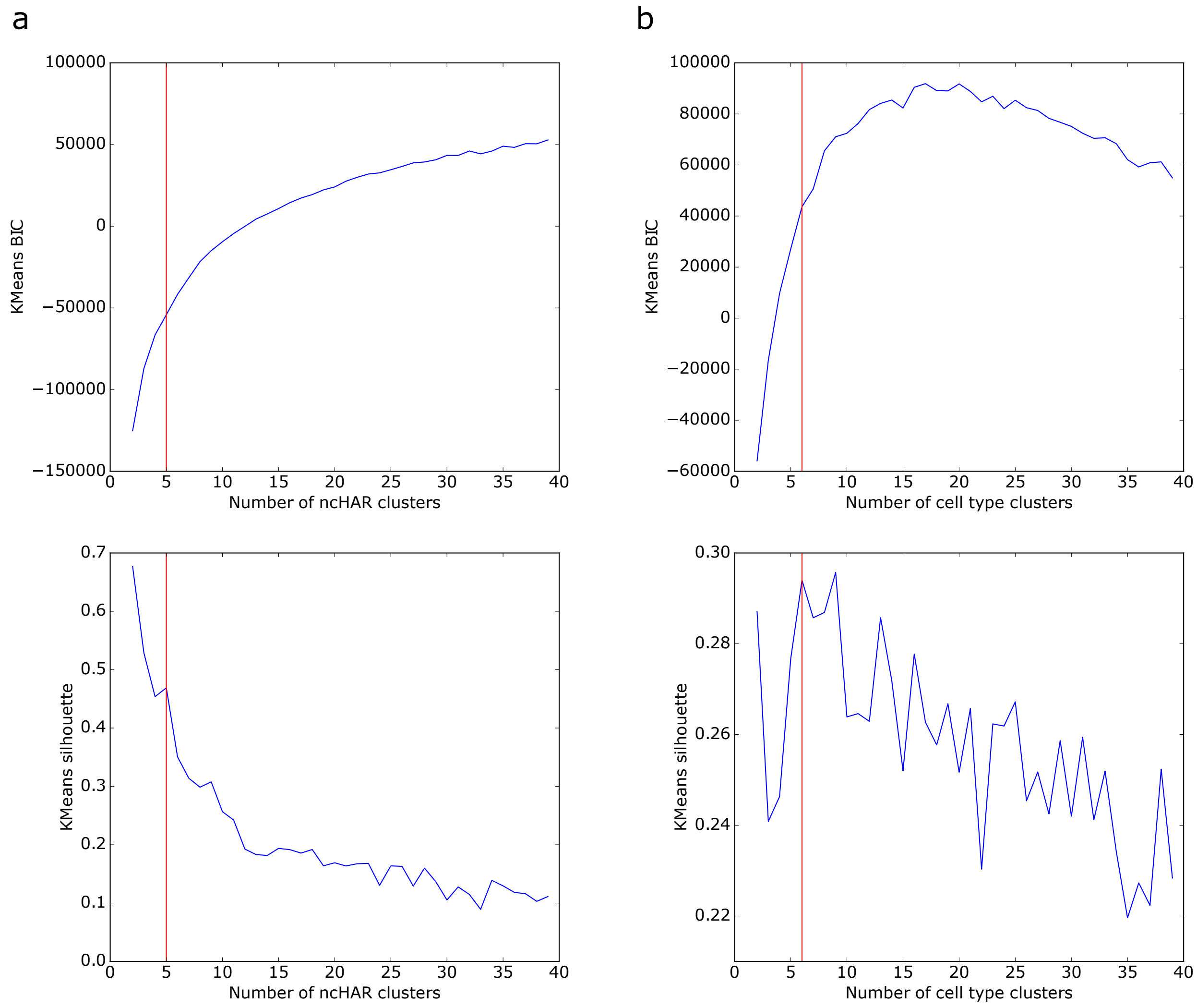

Supplementary Figure 16: **Elbow and silhouette analysis of imputed enhancer mark data over ncHARs supports the a choice of 5 ncHAR clusters and 6 cell type clusters.** To pick the number of ncHAR and cell type clusters, we used k-means clustering on the rows (ncHARs) and columns (cell types) of the biclustering input matrix (see Methods) for imputed data and conducted a Bayesian Information Criterion (BIC) "elbow" analysis, as well as a silhouette score analysis. Assessment of the quality of the clustering is based on finding a balance that achieves an appropriate number of clusters that are still well-separated. Elbow analysis based on BIC, as well as silhouette analysis suggests that, **a.** 5 is a reasonable number of clusters for ncHARs, and **b.** 6 is a reasonable number for cell types. The chosen cluster number is indicated by a vertical red line. In both cases the number of clusters is near the maximum of the second derivative in the elbow plots and also at a local maximum in the silhouette plots.

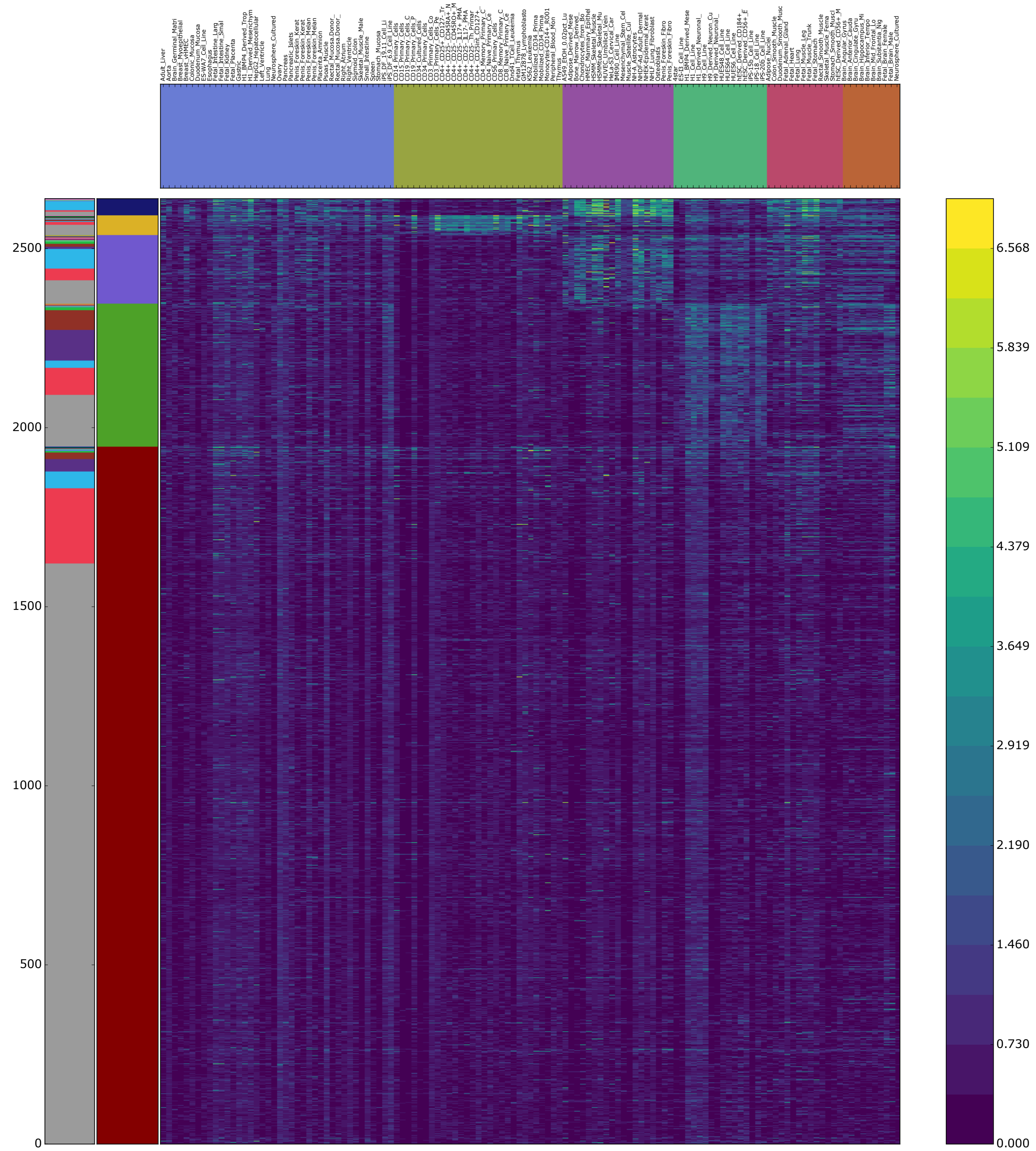

Supplementary Figure 17: **Heatmap showing biclustering results and signal from observed data at ncHARs for the H3K27ac, H3K4me1, and DNase assays.** The clustering results are very similar to those for imputed data reported in Fig. 5a.

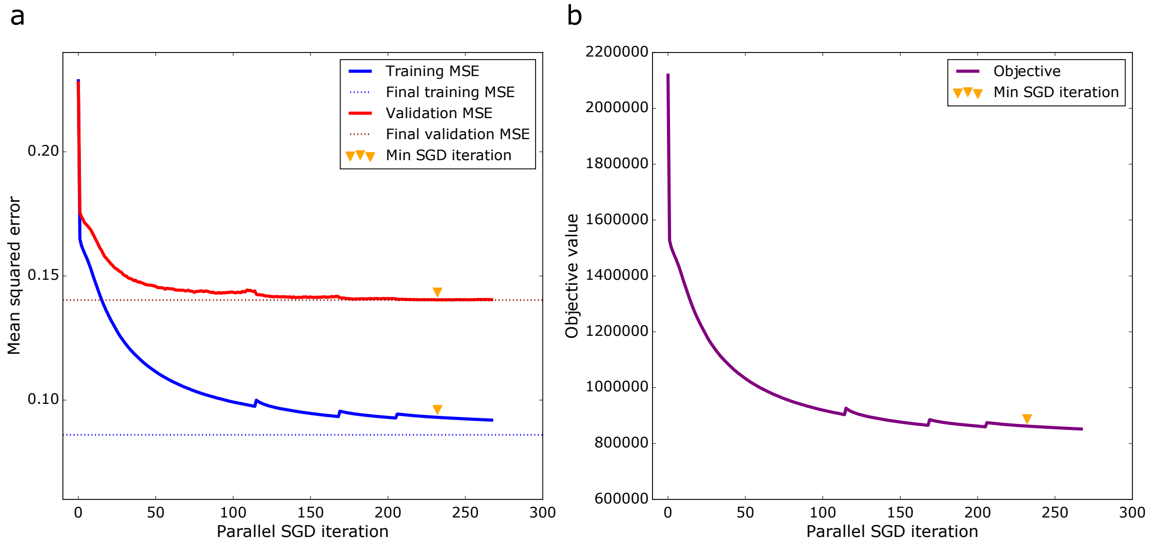

Supplementary Figure 18: **Training is halted before validation error increases.** **a.** Training and validation MSE as a function of the number of parallel SGD iterations during PREDICTD training. The initial vertical drop in the error corresponds to the burn-in phase of training before the parallel SGD iterations begin. The jags in the error curves indicate where the stopping criterion was met, so the training procedure reset the parameters to their values from the iteration with the previous minimum validation MSE, halved the learning rate, and continued training. Orange triangles indicate where the minimum validation MSE was achieved during parallel SGD, and the dotted lines indicate the model training and validation MSE after the final second order update of the genome parameters. The second order update decreased the validation MSE from 0.14034 to 0.14026. **b.** A similar plot of the objective value as a function of parallel SGD iterations shows that the objective value decreases and follows the same trend as the training error.

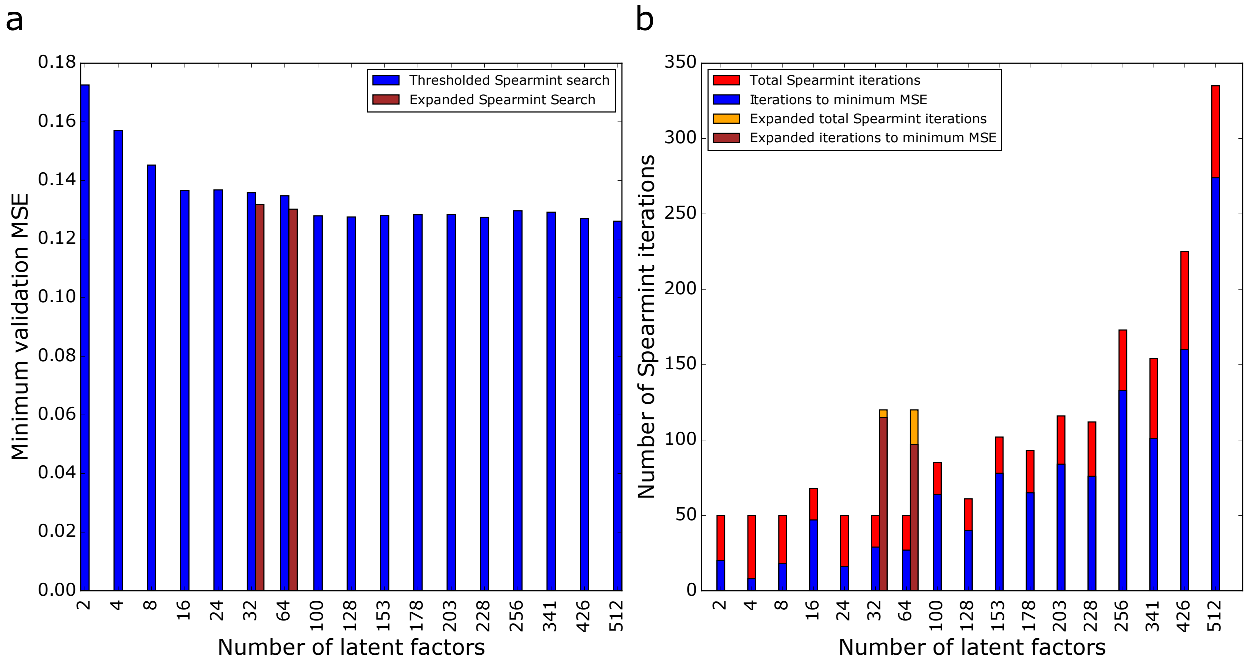

Supplementary Figure 19: **An extensive hyperparameter search supports selecting 100 latent factors as the model dimensionality for maximizing imputation performance. a.** Minimum validation error MSE for the best models found in each of 17 different Spearmint hyperparameter searches with different numbers of latent factors. The minimum validation MSE decreases as a function of increasing latent factor number until about 100 latent factors, suggesting that this dimensionality maximizes model performance while minimizing the redundancy of latent factors. Furthermore, if we allow the Spearmint hyperparameter search to continue until 120 iterations for 32 and 64 latent factors, we see that the 100 latent factor setting still finds a lower validation MSE. **b.** We required hyperparameter searches to get longer as the model dimensionality increased to allow sufficient time for Spearmint to search the solution spaces that become correspondingly more complex. We trained each level of latent factors for at least 50 Spearmint iterations or 40% of the number of latent factors, whichever was more, and only stopped Spearmint after it had additionally trained at least 20 iterations or 15% of the number of latent factors, whichever was more, past its best result (blue/red bars). We expanded the Spearmint search to 120 iterations for the 32 and 64 latent factor settings (orange/brown bars) to better resolve the solution space for the models that were only slightly less complex than our chosen setting of 100 latent factors.

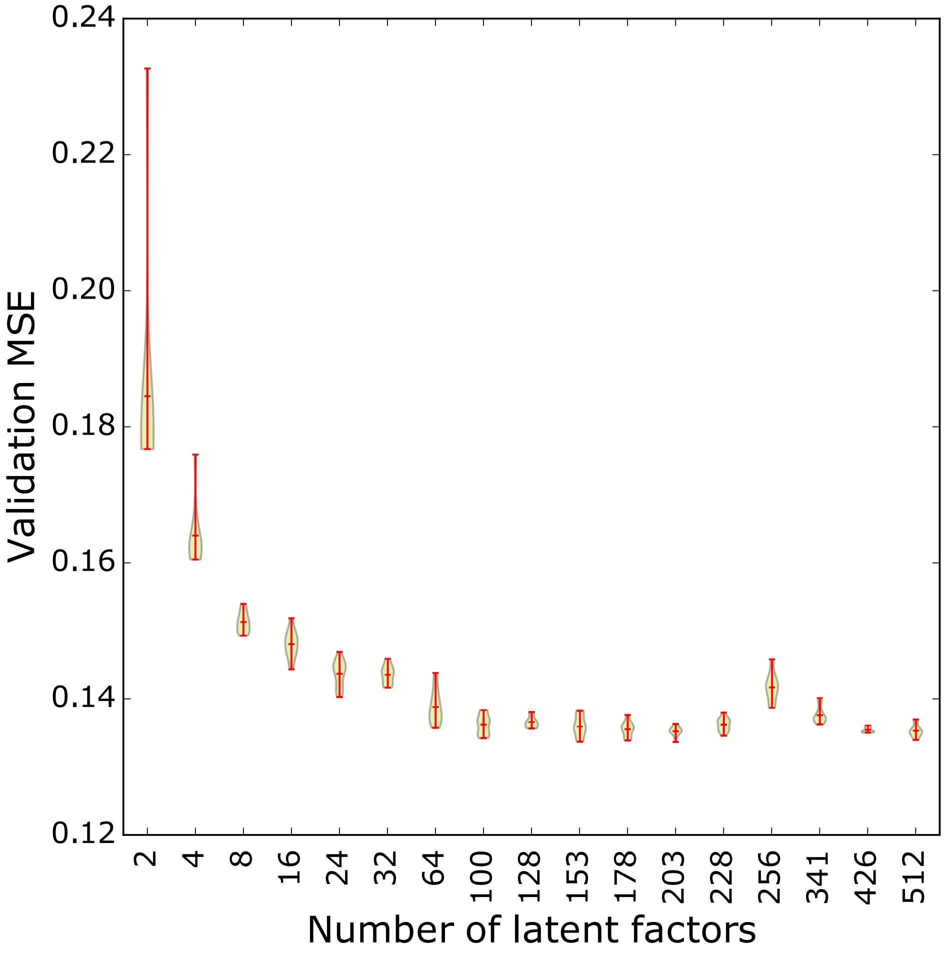

Supplementary Figure 20: **Training multiple models with different random initializations for each latent factor setting confirms the choice of 100 latent factors.** After doing an extensive hyperparameter search at 17 different latent factor settings (Supplementary Fig. 19), we used the same training/validation sets and the same genomic positions for training all models, and trained ten models with different random seeds for the best hyperparameter settings for each latent factor setting. For the 32 and 64 latent factor models, we used the hyperparameter settings from the expanded Spearmint search (Supplementary Fig. 19). The 64 latent factor distribution is greater than the 100 latent factor distribution by the Wilcoxon rank sum test with $p < 0.05$. The violin plots show the distribution of validation MSE for the ten trained models at each latent factor setting, with a horizontal line at the mean and whiskers indicating the extrema.

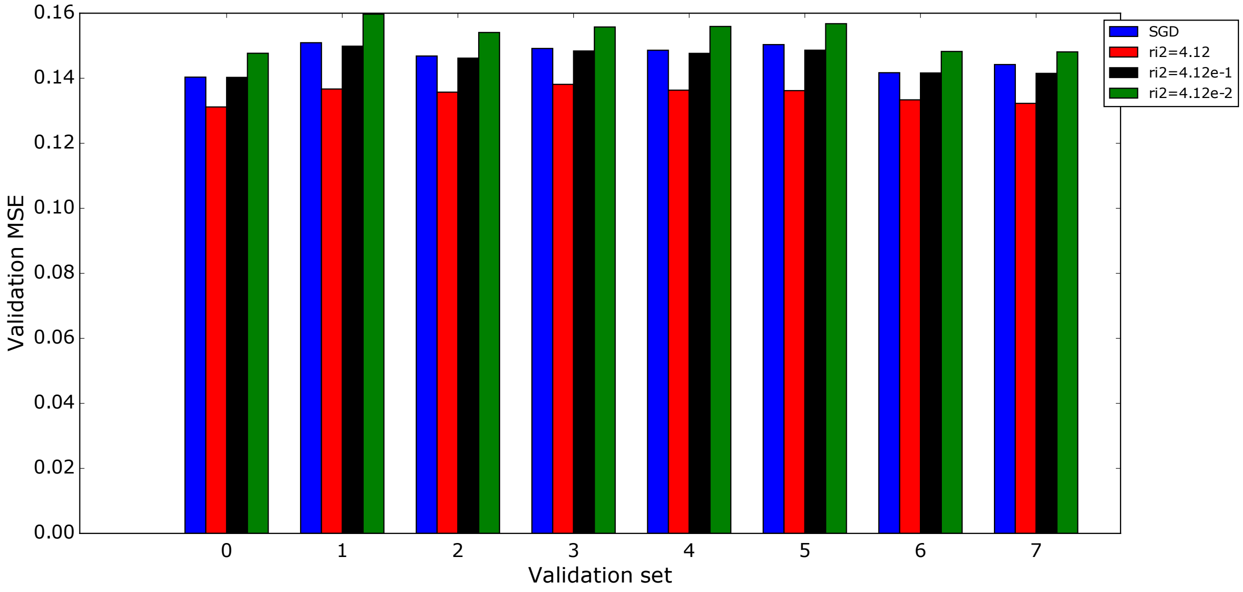

Supplementary Figure 21: **Averaging models provides additional regularization that can be balanced by reducing the regularization of the second order genome update.** The validation set MSE for the eight different validation folds corresponding to test set 0 after the second order genome update with different values of $\lambda_{\mathbf{G}_2}$. We chose $\lambda_{\mathbf{G}_2} = 0.412$ (black bars) as a value that imposed approximately the same amount of regularization as the stochastic gradient descent phase of training (blue bars) to avoid having too much regularization when we averaged the eight models together to calculate our final imputed values for the test set.
