# Supplementary material for: PREDICTD PaRallel Epigenomics Data Imputation with Cloud-based Tensor Decomposition
Source: Nat Commun. 2018 Apr 11;9:1402. doi: 10.1038/s41467-018-03635-9 (PMC5895786; doi:10.1038/s41467-018-03635-9)
Supplement: Supplementary file 20 — Description of Additional Supplementary Files(PDF 179 kb) [file 41467_2018_3635_MOESM20_ESM.pdf]

Description of Additional Supplementary Files

File Name: Supplementary Data 1
Description: Correlation and log ratio for all quality measures

File Name: Supplementary Data 2
Description: Cell type clusters from spectral biclustering of imputed and observed data

File Name: Supplementary Data 3
Description: ncHAR clusters from spectral biclustering of imputed and observed data

File Name: Supplementary Data 4
Description: ncHARs with validated enhancer activity from the literature

File Name: Supplementary Data 5
Description: GREAT analysis results for ncHARs from the "No Signal" cluster, whole genome background

File Name: Supplementary Data 6
Description: GREAT analysis results for ncHARs from the "Brain/ES" cluster, whole genome background

File Name: Supplementary Data 7
Description: GREAT analysis results for ncHARs from the "Brain/ES" cluster, all ncHARs background

File Name: Supplementary Data 8
Description: GREAT analysis results for ncHARs from the "Epithelial/Mesenchymal" cluster, whole genome background

File Name: Supplementary Data 9
Description: GREAT analysis results for ncHARs from the "Epithelial/Mesenchymal" cluster, all ncHARs background

File Name: Supplementary Data 10
Description: GREAT analysis results for ncHARs from the "Non-immune" clus-ter, whole genome background

File Name: Supplementary Data 11
Description: GREAT analysis results for ncHARs from the "Non-immune" clus-ter, all ncHARs background

File Name: Supplementary Data 12
Description: GREAT analysis results for ncHARs from the "Immune" cluster, whole genome background

File Name: Supplementary Data 13

Description: PREDICTD quality measures by experiment

File Name: Supplementary Data 14
Description: Main Effects quality measures by experiment

File Name: Supplementary Data 15
Description: ChromImpute quality measures by experiment

File Name: Supplementary Data 16
Description: PREDICTD and ChromImpute Average Model quality measures by experiment

File Name: Supplementary Data 17
Description: ENCODE accession identifiers for PREDICTD imputed data sets
